# Improvement in delivery of type 2 diabetes services differs by mode of care: a retrospective longitudinal analysis in the Aboriginal and Torres Strait Islander Primary Health Care setting

**DOI:** 10.1186/s12913-016-1812-9

**Published:** 2016-10-07

**Authors:** Gill Schierhout, Veronica Matthews, Christine Connors, Sandra Thompson, Ru Kwedza, Catherine Kennedy, Ross Bailie

**Affiliations:** 1 Menzies School of Health Research, Brisbane, Australia; 2 The Kirby Institute, University of New South Wales, Sydney, Australia; 3 Department of Health, Darwin, NT Australia; 4 Western Australian Centre for Rural Health, University of Western Australia, Geraldton, WA Australia; 5 Queensland Health, Cairns, QLD Australia; 6 Maari Ma Health Aboriginal Corporation, Broken Hill, Far West New South Wales Australia; 7 University Centre for Rural Health, University of Sydney, Lismore, NSW Australia

**Keywords:** Health systems, Quality improvement, Primary care, Impact, Clinical guidelines

## Abstract

**Background:**

Addressing evidence-practice gaps in primary care remains a significant public health challenge and is likely to require action at different levels of the health system. Whilst Continuous Quality Improvement (CQI) is associated with improvements in overall delivery, little is known about delivery of different types of care processes, and their relative improvement during CQI.

**Methods:**

We used data from over 15,000 clinical audit records of clients with Type 2 diabetes collected as part of a wide-scale CQI program implemented between 2005 and 2014 in 162 Aboriginal and Torres Strait Islander health centres. We abstracted data from clinical records on 15 service items recommended in clinical guidelines and categorised these items into five modes of care on the basis of the mechanism through which care is delivered: laboratory tests; generalist-delivered physical checks; specialist-delivered checks; education/counselling for nutrition and physical activity and education/counselling for high risk substance use. We calculated delivery for each patient for each of mode of care by determining the proportion of recommended services delivered for that mode. We used multilevel regression models to quantify variation attributable to health centre or client level factors and to identify factors associated with greater adherence to clinical guidelines for each mode of care.

**Results:**

Clients on average received 43 to 60 % of recommended care in 2005/6. Different modes of care showed different patterns of improvement. Generalist-delivered physical checks (delivered by a non-specialist) showed a steady year on year increase, delivery of laboratory tests showed improvement only in the later years of the study, and delivery of counselling/education interventions showed early improvement which then plateaued. Health centres participating in CQI had increased odds of top quartile service delivery for all modes compared to baseline, but effects differed by mode. Health centre factors explained 20–52 % of the variation across jurisdictions and health centres for different modes of care.

**Conclusions:**

Levels of adherence to clinical guidelines and patterns of improvement during participation in a CQI program differed for different modes of care. Policy and funding decisions may have had important effects on the level and nature of improvements achieved.

## Background

The optimal delivery of evidence based practice in chronic disease management is challenging in primary health care (PHC) and evidence-practice gaps have been documented in PHC systems internationally. PHC delivery for Aboriginal and Torres Strait Islander people with chronic disease follows this pattern, but little is known about how to design and target interventions to achieve system-wide population level improvement in chronic disease outcomes. We have previously reported overall improvements in delivery of Type 2 diabetes services to Aboriginal and Torres Strait Islander communities during health centre participation in a wide-scale Continuous Quality Improvement (CQI) program, with duration of CQI participation associated with higher overall service delivery [1].

Whilst overall measures of service delivery provide a summary of the evidence- practice gap across the scope of recommended services, they lack the granularity needed to identify the range and nature of potential system-wide solutions to problems of care quality [2]. McGlynn and colleagues [2] have suggested that understanding performance in relation to *how* care is delivered, for example, distinguishing between care processes that are delivered through laboratory tests, physical checks, prescription of medications, and so on, may provide insight into the kinds of higher level system changes needed to improve quality. The assumption is that care processes delivered through different mechanisms (referred to as ‘modes of care’ [2]) have differing system requirements. With increasing availability of data on PHC performance internationally, it is timely to explore the utility and robustness of different forms of data disaggregation and analysis to support system improvement.

A range of health centre and individual level factors contribute to explaining variation in the overall quality of care received by clients attending PHC services [1, 3, 4]. Do the same factors explain variation for different categories of care processes, or do different factors come into play depending on the mechanisms through which care is delivered? Drawing on data available from 9 years of implementation of a wide- scale CQI program in Aboriginal and Torres Strait Islander PHC, and with a focus on patients with Type 2 diabetes, this study explores the variation and extent of improvement of five different modes of care over time. Specifically we address several unanswered questions:

- How much variation is there in delivery of care according to mode?
- Do all modes of care improve over time and with duration of participation in CQI?
- Do the same health centre and individual client characteristics that influence improvements overall, and in one mode of care, similarly influence improvements in other modes?

This paper contributes to efforts to interpret and use aggregate CQI data to identify which aspects of the health system need strengthening to support improved service delivery.

## Methods

### Study context and design

This is an analysis of longitudinal data derived from use of standard clinical audit tools and protocols (including sampling processes) from health centres participating in a wide scale CQI program that has been operating in Aboriginal and Torres Strait Islander PHC settings for over a decade.

Aboriginal and Torres Strait Islander people in Australia access PHC in three major services sectors: Aboriginal community controlled health services, state and territory funded/operated health services, and general practice. Aboriginal community controlled health centres, and state and territory funded/operated centres, and their service populations are the setting for this project. These health centres provide PHC predominantly, although not exclusively to Aboriginal and Torres Strait Islander people. They are at the forefront of providing PHC to Aboriginal and Torres Strait Islander communities particularly in rural and remote settings where there are relatively few medical practitioners. Health centres range in size from small service centres (where in some cases the regular staff consists of only a single nurse, with other staff providing services through regular visits), through to large centres staffed by a range of health professionals. In this context, a wide-scale CQI initiative, “the ABCD CQI project”, employs a systems approach to improving PHC delivery through evidence-based clinical audit and systems assessment tools and processes. The project was developed through participatory action research approaches, commencing with 12 remote health centres in the Northern Territory in 2002, expanding to around 220 health centres across five jurisdictions by September 2014 (with 175 involved in research activities).

At the health centre level, centres participating in the ABCD CQI project conduct annual audits of their care quality using standardised tools and audit processes developed for this purpose. Participating centres have access to a web-based platform, which provides real-time analysis of their audit data, automated reporting, and ability to compare their services with other de-identified services. These functions are supported by a not-for-profit service agency, One21seventy [5] and integrated into support provided by CQI facilitators in some jurisdictions. At the regional and national level, the ABCD National Research Partnership (2010–2014) provides mechanisms for health centre and service managers, service providers and policy makers to articulate their priorities related to the research component of the project, and contribute to interpretation of aggregate CQI data with the aim of identifying factors underlying regional and national variation in quality of care.

A suite of tools is available through the project, covering different aspects of PHC service delivery, including vascular and metabolic disease, maternal and child health, preventive health, mental health, and health promotion. This study focused on services provided to patients with Type 2 diabetes.

### Data sources

One hundred and sixty two health centres participating in the ABCD project were included in the study. These were all the participating health centres that had conducted clinical audits of patients with diagnosed Type 2 diabetes between July 2005 and September 2014, using the ABCD/One21seventy audit tools and processes. Owing to the continued incremental engagement in the project, there are more health centres and patients included in this analysis compared to our previously published longitudinal analysis [1] that included data collected to the end of 2012. Eligible clients are those aged 16 years and older with a documented diagnosis of Type 2 diabetes who have lived in the community serviced by the health centre for a minimum of 6 of the preceding 12 months. For health services with more than 30 eligible clients, the protocol recommends and provides guidance for health centres to draw a sufficient number of records to achieve a precision of 90 or 95 % confidence of the sample representing the population [5]. Where health services have 30 or fewer clients meeting eligibility criteria, records of all eligible clients are audited. This sampling approach yielded a total sample of 15 622 audited records available for analysis.

### Measures

To assess Type 2 diabetes service delivery, the study measured 15 service items from best practice guidelines across the states and territories [6–8] (Table 1). We classified the service items according to the mode of delivery: laboratory tests; generalist physical checks (those typically delivered by members of the primary health care team); specialist-delivered checks (including retinal checks typically delivered by an optometrist or ophthalmologist and foot checks delivered by a podiatrist); counselling for risk factors (nutrition and physical exercise), and counselling for tobacco or alcohol use (for known high risk users of tobacco and alcohol). The classification of service items into relevant categories was done in consultation with service providers and policy makers during bi-annual meetings held as part of the ABCD National Research Partnership project. There was some modification in initial categories proposed, reflecting service providers‘ and managers’ perspectives of the system requirements for delivery of the service items. Recommendations included separating specialist-delivered service items from those items delivered by the PHC team, and separating counselling type interventions that focused on high risk substance users, from counselling type interventions to address nutrition and physical activity.

**Table 1:** Recommended schedule for Type 2 diabetes patients by mode of care [6, 7]

	Mode of care/service item	Frequency
Services for all type 2 diabetes clients	Laboratory Tests	
	ACR	Yearly
	eGFR	Yearly
	Lipids	Yearly
	HbA1c	3 monthly
	Generalist Physical Checks	
	Weight	6 monthly
	Waist circumference	6 monthly
	BMI	Yearly
	Blood Pressure	3–6 monthly
	Visual acuity	Yearly
	Specialist Physical Checks	
	Dilated eye check	Yearly
	Foot check	Yearly
	Counselling	
	Nutrition	Yearly
	Physical activity	Yearly
High risk substance use	Counselling	
	Smoking	Yearly
	High risk alcohol consumption	Yearly

Health centre characteristics included duration of participation in the ABCD CQI projects, size of population served, governance (whether community controlled or government operated) and location (remote or non-remote). For each health centre, we calculated the proportion of diabetes patients who had not attended in the 6 months prior to audit and created a binary variable using a cut off at the 75^th^ percentile.

At the patient level, demographic characteristics (sex, age, Indigenous status) were extracted from clinical records. Documented chronic health conditions (diabetes, hypertension, chronic heart disease and chronic kidney disease) were recorded as present or absent and the number of co-morbidities was calculated by summing the record of presence of each condition. Similarly, documented complications (retinopathy, neuropathy, foot ulcers and amputations) were recorded as present or absent, and number of complications calculated per individual.

### Analysis

We used STATA software, V.13 for statistical analysis. Composite scores for each mode of care for each patient were calculated by dividing the sum of the services delivered by the number of services for which that person was eligible. Table 1 provides a list of the service items used to construct the composite scores for each mode of care. Composite scores were then aggregated for each health centre (“aggregate scores”). At the health centre level, a mean adherence to delivery of recommended services in a given health service represented an overall performance score for that health centre for that mode of care in a given audit cycle. Each aggregate score was converted into a binary variable that categorized ‘higher’ performance as being within the top quartile (75–100 %) of delivery across all health centres measured at baseline for that mode of care. ‘Lower performance’ was categorised as being below the top quartile.

We used multi-level mixed effects logistic regression models to quantify the variation in service delivery separately for each mode of care. These models allowed for the hierarchical structure of the data (patients nested within health centres, nested within jurisdictions). We calculated crude odds ratios to measure the unadjusted association between each mode of care and health service and client characteristics. Potential interactions were checked for significance, and significant interaction terms included in the adjusted models as explained below.

We adopted a stepwise modelling strategy starting with Model A that included the audit year variable only, to test the influence of jurisdictions and health centres on service delivery over time. Health centre (Model B) and then patient level variables (Model C) from the unadjusted analyses were introduced into the empty model.

The reduction in variance due to the stepwise introduction of the different variables in the models was determined by the proportional change in variance (PCV) at different levels. The PCV provides an estimate of the extent to which health centre level factors and individual patient characteristics may explain individual differences in propensity for better delivery of health care [8, 9]

We calculated median odds ratios (MORs) to help interpret variance in the odds ratio scale. The MOR describes the increased (median) probability of receiving ‘a high proportion of recommended care processes if a patient was to change health centre (or jurisdiction) [10]. If the MOR was equal to 1, there would be no difference between health centres in their probability of adhering to the recommended care processes. If there were important differences between health centres, the MOR would be large. The accuracy of the variance estimates was evaluated by their standard error (SE). A *p*-value <0.05 was considered significant.

### Ethics

Ethics approval was obtained from research ethics committees in each jurisdiction (Human Research Ethics Committee of the Northern Territory Department of Health and Menzies School of Health Research (HREC-EC00153); Central Australian Human Research Ethics Committee (HREC-12-53); New South Wales Greater Western Area Health Service Human Research Committee (HREC/11/GWAHS/23); Queensland Human Research Ethics Committee Darling Downs Health Services District (HREC/11/QTDD/47); South Australian Aboriginal Health Research Ethics Committee (04-10-319); Curtin University Human Research Ethics Committee (HR140/2008); Western Australian Country Health Services Research Ethics Committee (2011/27); Western Australia Aboriginal Health Information and Ethics Committee (111-8/05); University of Western Australia Human Research Ethics Committee (RA/4/1/5051)).

## Results

Of the 162 participating health centres with available diabetes audit data, 73 % were located in remote areas, 46 % had a service population of 500 or fewer, and 39 % had participated in the ABCD CQI project for three or more years (Table 2). In all jurisdictions, on average, over 90 % of clients audited had attended the health centre within the previous 6 months. From these health centres, records of 15,622 clients with a diagnosis of Type 2 diabetes were audited between 1 July 2005 and September 2014 (Table 3). For analysis purposes, audits conducted at the start of the project in 2005 (315 audits from 12 health centres) were merged into the 2006 audit year. The mean age of clients was 53 years and 57 % were women. Over 90 % of records from the Northern Territory, Western Australia and South Australia were for Aboriginal or Torres Strait Islander people, compared to 76 % in Queensland and 55 % in Far West New South Wales. Around 16 % of clients had one or more diabetes complications, and 35 % (*n* = 5476) were recorded as having high risk tobacco or alcohol use.

**Table 2:** Characteristics of participating health centres by jurisdiction

		Far West New South Wales	Norther Territory	Queensland	South Australia	Western Australia	Total
Total number health centres		6	65	69	8	14	162
Location	Remote	3 (50)	61 (94)	46 (67)	2 (25)	7 (50)	119 (73)
	Non-remote	3 (50)	4 (6)	23 (33)	6 (75)	7 (50)	43 (27)
Governance	Community-controlled	6 (100)	19 (29)	1 (1)	5 (63)	8 (57)	39 (24)
	Government	0	46 (71)	68 (99)	3 (37)	6 (43)	123 (76)
Service Population	<501	2 (33)	36 (55)	34 (49)	1 (12)	2 (14)	75 (46)
	501–999	1 (17)	12 (18)	14 (20)	3 (38)	0	30 (19)
	>999	3 (50)	17 (26)	21 (30)	4 (50)	12 (86)	57 (35)
Mean % (and range) clients attending last 6 months		79	96	90	83	83	91
		(53–97)	(77–100)	(60–100)	(62–100)	(50–100)	(50–100)
Duration of participation in ABCD CQI	<1 year	0	9 (13)	14 (20)	2 (25)	3 (21)	28 (17)
	1–2 years	0	27 (42)	32 (46)	5 (63)	7 (50)	71 (44)
	3 or more years	6 (100)	29 (45)	23 (33)	1 (12)	4 (29)	63 (39)

**Table 3:** Patient characteristics by jurisdiction (*N* and % unless otherwise indicated)

		Far West NSW	NT	QLD	SA	WA	Total
Total number health centres		6	65	69	8	14	162
Location	Remote	3 (50)	61 (94)	46 (67)	2 (25)	7 (50)	119 (73)
	Non-remote	3 (50)	4 (6)	23 (33)	6 (75)	7 (50)	43 (27)
Governance	Community-controlled	6 (100)	19 (29)	1 (1)	5 (63)	8 (57)	39 (24)
	Government	0	46 (71)	68 (99)	3 (37)	6 (43)	123 (76)
Service Population	<501	2 (33)	36 (55)	34 (49)	1 (12)	2 (14)	75 (46)
	501–999	1 (17)	12 (18)	14 (20)	3 (38)	0	30 (19)
	>999	3 (50)	17 (26)	21 (30)	4 (50)	12 (86)	57 (35)
Mean % (and range) clients attending last 6 months		79	96	90	83	83	91
		(53–97)	(77–100)	(60–100)	(62–100)	(50–100)	(50–100)
Duration of participation in ABCD CQI	<1 year	0	9 (13)	14 (20)	2 (25)	3 (21)	28 (17)
	1–2 years	0	27 (42)	32 (46)	5 (63)	7 (50)	71 (44)
	3 or more years	6 (100)	29 (45)	23 (33)	1 (12)	4 (29)	63 (39)

**Fig. 1:**
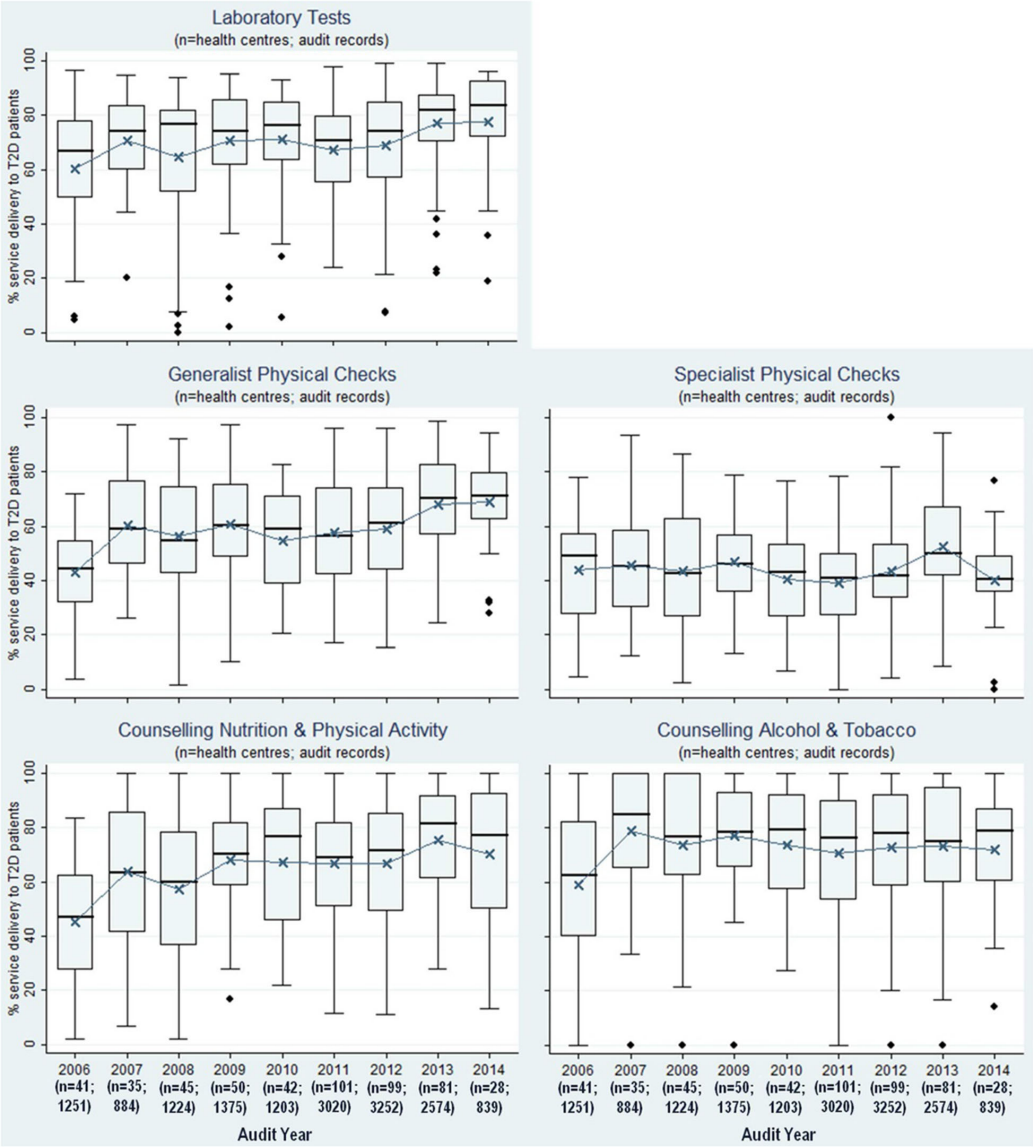
Type 2 diabetes service delivery over time by mode of care—(mean percent of the aggregate composite scores for each health centre for each mode of care)

The mean percent delivery across health centres for different modes of care in 2005/6 ranged from 43 % for generalist physical checks to 60 % for laboratory tests (Fig. 1). Wide variation in delivery of each mode of care was evident across all health centres within an audit year and over time (Fig. 1). The mean percent delivery of all modes of care with the exception of specialist-delivered checks was higher in 2014 than in 2005/6.

The unadjusted logistic regression analysis results (Table 4) show strong effects of audit year for some modes of care, but not for others. For generalist physical checks there was a steady increase in the odds of health centres being within the top quartile of performance from baseline to 2014. Health centres had approximately 9 times greater odds of top quartile performance for generalist physical checks in 2014 compared to 2005/6 (95 % CI 7.14–12.34), with a steady year-on-year increase in the effect size (Table 4).

**Table 4:** Unadjusted multilevel logistic regression models of health centre and client level factors associated with top quartile delivery of guideline-scheduled services

		Laboratory tests^a^	Generalist physical checks^a^	Specialist physical checks^a^	Counselling or education (nutrition and physical activity)^a^	Counselling or education (high risk alcohol or tobacco use)^b^
	Fixed effects	OR (95 % CI)	ORs (95 % CI)	ORs (95 % CI)	ORs (95 % CI)	ORs (95 % CI)
Audit Year	2005/2006	1.00 (reference)	1.00 (reference)	1.00 (reference)	1.00 (reference)	1.00 (reference)
	2007	0.85 (0.71–1.05)	2.45 (1.98–3.03)**	0.88 (0.72–1.08)	1.68 (1.38–2.04)**	3.30 (2.23–4.88)**
	2008	0.87 (0.72–1.04)	3.20 (2.61–3.93)**	0.95 (0.80–1.13)	1.69 (1.41–2.01)**	2.32 (1.67–3.21)**
	2009	1.08 (0.90–1.29)	5.09 (4.15–6.25)**	1.26 (1.04–1.51)*	2.69 (2.24–3.23)**	2.33 (1.71–3.18)**
	2010	1.23 (1.02–1.49(*	5.11 (4.10–6.36)**	1.06 (0.87–1.29)	2.69 (2.22–3.26)**	2.21 (1.59–3.09)**
	2011	0.81 (0.69–0,97)**	5.12 (4.19–6.2)**	1.02 (0.85–1.21)	2.14 (1.80–2.54)**	2.25 (1.67–3.02)**
	2012	1.26 (1.05–1.50)*	6.65 (5.37–8.04)**	1.29 (1.08–1.53)**	2.18 (1.83–2.59)**	2.25 (1.67–3.02)**
	2013	1.67 (1.40–2.00)**	9.77 (7.92–12.03)**	1.64 (1.36–1.97)**	2.43 (2.36–4.12)**	2.76 (2.08–3.76)**
	2014	1.82 (1.45–2.28)**	9.57 (7.41–12.34)**	1.65 (1.30–2.08)**	2.48 (1.98–3.12)**	2.41 (1.67–3.45)**
*Health Centre Characteristics*						
Duration of CQI participation	Baseline	1.00 (reference)	1.00 (reference)	1.00 (reference)	1.00 (reference)	1.00 (reference)
	1–2 cycles	1.47*1.35–1.61)**	2.40 (2.17–2.65)**	1.40 (1.28–1.53)**	1.89 (1.73–2.07)**	1.59 (1.36–1.85)**
	≥3 cycles	1.54 (1.39–1.72)**	4.13 (3.66–4.66)**	1.48 (1.33–1.65)**	1.79 (1.61–2.00)**	1.77 (1.52–2.07)**
Location of health centre	Non-remote	1.00 (reference)	1.00 (reference)	1.00 (reference)	1.00 (reference)	
	Remote	4.01(2.84–5.68)**	1.85 (1.25–2.74)**	1.66 (1.25–2.22)**	1.82 (1.26–2.63)**	1.16 (0.87–1.56)
Governance	Community-Controlled	1.00 (reference)	1.00 (reference)	1.00 (reference)	1.00 (reference)	1.00 (reference)
	Government operated	0.85 (0.54–1.33)	0.99 (0.64–1.54)	1.03 (0.74–1.43)	1.18 (0.80–1.75)	0.75 (0.57–0.98)*
Service population	≤500	1.00 (reference)	1.00 (reference)	1.00 (reference)	1.00 (reference)	1.00 (reference)
	>500– < 1000	1.08 (0.67–1.61)	1.00 (0.65–1.54)	0.88 (0.64–1.20)	0.89 (0.60–1.32)	0.94 (0.67–1.31)
	≥1000	0.71 (0.49–1.04)	0.66 (0.45–0.96)	0.71 (0.54–0.93)	0.68 (0.48–0.97)	1.01 (0.76–1.34)
% Clients attending in past 6 months	<97 %	1.00 (reference)	1.00 (reference)	1.00 (reference)	1.00 (reference)	1.00 (reference)
	≥97 %	1.59 (1.44–1.75)**	1.73 (1.56–1.92)**	1.61 (1.45–1.78)**	1.48 (1.34–1.64)**	1.13 (0.96–1.32)
*Client Characteristics*						
Sex	Male	1.00 (reference)	1.00 (reference)	1.00 (reference)	1.00 (reference)	1.00 (reference)
	Female	1.08 (0.97–1.11)	0.99 (0.92–1.17)	1.09 (1.02–1.17)	1.00 (0.95–1.07)	0.99 (0.74–1.12)
Age	≥15- < 25	1.00 (reference)	1.00 (reference)	1.00 (reference)	1.00 (reference)	1.00 (reference)
	≥25- < 40	1.20 (0.91–1.57)	1.08 (0.81–1.45)	1.51 (1.15–1.96)**	1.37 (1.05–1.79)**	0.94 (0.59–1.48)
	≥40- < 55	1.45 (1.11–1.88)	1.50 (1.13–1.99)**	2.26 (1.74–2.92)**	1.58 (1.12–2.04)**	1.05 (0.67–1.65)
	≥55	1.62 (1.24–2.11)**	1.62 (1.22–2.15)**	3,27 (2,52–4.24)**	1.57 (0.53–1.34)	1.00 (0.63–1.58)
Indigenous Status	Non-Indigenous	1.00 (reference)	1.00 (reference)	1.00 (reference)	1.00 (reference)	1.00 (reference)
	Indigenous	1.11 (0.94–1.30)	1.35 (1.14–1.60)**	1.15 (0.98–1.35)	1.02 (0.87–1.19)	1.17 (0.84–1.64)
Comorbidity	None	1.00 (reference)	1.00 (reference)	1.00 (reference)	1.00 (reference)	1.00 (reference)
	One or more	1.53 (1.41–1.67)**	1.52 (1.39–1.66)**	1.65 (1.52–1.79)**	1.46 (1.35–1.58)**	1.24 (1/11–1.39)**
Complications	None	1.00 (reference)	1.00 (reference)	1.00 (reference)	1.00 (reference)	1.00 (reference)
	1–2 complications	1.27 (1.14–1.42)**	1.25 (1.11–1.40)**	2.62 (2.30–1.40)**	1,24 (1.11–1.39)**	1.12 (0.92–1.38)
	>2 complications	1.35 (1.13–1.61)**	1.00 (0.82–1.20)	3.47 (2.76–4.35)**	1.08 (0.91–1.30)	1.19 (0.86–1.66)

^a^ *N* = 15,622 clients and 162 health centres

^b^ *N* = 5962 clients and 162 health centres

*p < 0.05

**p = <0.01

There were modest effects of audit year evident for laboratory tests and for counselling/education modes of care, with no consistent pattern of increase in effect sizes in later audit years relative to 2005/6 for these measures. For laboratory tests, the first audit year in which health centres had an increased odds of being within the top quartile of performance compared to2005/6, was in 2010, with increasing effect sizes in years following 2011 in the unadjusted analysis. For counselling/education modes of care, health centres had approximately 2–3 times the odds of being within the top quartile of performance in 2007 compared to baseline, but odds did not increase in subsequent years. Differences in service delivery in audit years relative to 2005/6 diminished after adjusting for health centre and client level factors, including duration of participation in CQI, but were still significant (Tables 8 and 9, Models B and C). Delivery of specialist delivered checks in later audit years did not differ significantly from 2005/6 levels in the adjusted models (Tables 4 and 7). We did not conduct formal statistical tests of trends over time.

The PCV in Model B (Tables 5, 6, 7, 8 and 9) shows that the addition of health centre factors explained 52, 30, 34, 21 and 20 % of the variation across jurisdictions and health centres for laboratory tests, generalist physical checks, specialist-delivered checks, counselling/education for nutrition and physical activity and counselling/education for high risk substance use respectively.

**Table 5:** Adjusted multilevel logistic regression models of health centre and client level factors associated with top quartile delivery of guideline-scheduled laboratory tests (*N* = 15,622 clients, 162 health centres)

		Model A empty	Model B adjusted	Model C adjusted
	Fixed effects	OR (95 % CI)	ORs (95 % CI)	ORs (95 % CI)
Audit Year	2005/2006	1.00 (reference)	1.00 (reference)	1.00 (reference)
	2007	0.86 (0.71–1.05)	0.77 (0.62–0.95)*	0.80 (0.65–0.99)*
	2008	0.87 (0.72–1.04)	0.76 (0.61–0.93)**	0.80 (0.65–0.99)*
	2009	1.08 (0.90–1.29)	0.92 (0.74–1.14)	0.94 (0.75–1.17)
	2010	1.23 (1.02–1.49)*	1.03 (0.82–1.30)	1.03 (0.82–1.30)
	2011	0.81 (0.69–0.97)**	0.73 (0.58–0.92)**	0.72 (0.57–0.91)**
	2012	1.26 (1.05–1.50)*	1.01 (0.77–1.32)	0.99 (0.75–1.29)
	2013	1.67 (1.40–2.00)**	1.28 (0.96–1.70)	1.22 (0.92–1.63)
	2014	1.82 (1.45–2.28)**	1.49 (1.08–2.06)*	1.44 (1.04–1.99)*
*Health Centre Characteristics*				
Location X duration of CQI participation				
Non-remote	Baseline		1.00 (reference)	1.00 (reference)
	1–2 cycles		1.03 (0.82–1.28)	0.99 (0.79–1.23)
	≥3 cycles		0.91 (0.66–1.26)	0.87 (0.63–1.21)
Remote	Baseline		1.00 (reference)	1.00 (reference)
	1–2 cycles		4.43 (3.06–6.41)**	4.59 (3.17–6.63)**
	≥3 cycles		4.68 (3.07–7.14)**	4.84 (3.18–7.37)**
Governance	Community-Controlled		1.00 (reference)	1.00 (reference)
	Government operated		0.76 (0.53–1.08)	0.78 (0.55–1.11)
Service population	≤500		1.00 (reference)	1.00 (reference)
	>500- < 1000		1.24 (0.88–1.76)	1.23 (0.87–1.73)
	≥1000		1.16 (0.84–1.61)	1.17 (0.84–1.61)
% Clients attending in past 6 months	<97 %		1.00 (reference)	1.00 (reference)
	≥97 %		1.39 (1.26–1.54)**	1.38 (1.25–1.53)**
*Client Characteristics*				
Sex	Male			1.00 (reference)
	Female			1.06 (0.99–1.14)
Age	≥15- < 25			1.00 (reference)
	≥25- < 40			1.12 (0.85–1.47)
	≥40- < 55			1.27 (0.97–1.67)
	≥55			1.39 (1.06–1.82)*
Indigenous Status	Non-Indigenous			1.00 (reference)
	Indigenous			1.07 (0.91–1.26)
Comorbidity	None			1.00 (reference)
	One or more			1.38 (1.27–1.51)**
Complications	None			1.00 (reference)
	1–2 complications			1.18 (1.06–1.32)**
	>2 complications			1.24 (1.03–1.48)*
Random effects (intercepts)				
	State (variance (SE))	0.51 (0.37)	0.16 (0.13)	0.15 (0.12)
	MOR_STATE_	1.97	1.46	1.44
* PCV (% explained variance)*		-	*70 %*	*72 %*
Health Centre (variance (SE))		0.96 (0.14)	0.56 (0.09)	0.55 (0.09)
	MOR_HC_	2.55	2.04	2.02
* PCV (% explained variance)*		-	*42 %*	*43 %*
State & Health Centre (variance)		1.470	0.717	0.692
	MOR_STATE-HC_	3.18	2.24	2.21
* PCV (% explained variance)*		-	*52 %*	*53 %*
	Patient (variance (SE))	0.39 (0.14)	0.19 (0.06)	0.11 (0.03)

MOR (Median odds ratio): odds of receiving ‘top quartile’ service delivery if patient was to change health centre or jurisdiction; PCV (Proportional change in variance): percent variation explained in odds for better health care delivery by introduction of health centre or patient level factors

*p < 0.05

**p = <0.01

**Table 6:** Adjusted multilevel logistic regression models of health centre and client level factors associated with top quartile delivery of guideline-scheduled generalist physical checks (*N* = 15,622 clients, 162 health centres)

		Model A empty	Model B adjusted	Model C adjusted
	Fixed effects	OR (95 % CI)	ORs (95 % CI)	ORs (95 % CI)
Audit Year	2005/2006	1.00 (reference)	1.00 (reference)	1.00 (reference)
	2007	2.45 (1.98–3.03)**	1.75 (1.39–2.20)**	1.82 (1.45–2.29)**
	2008	3.20 (2.61–3.93)**	1.97 (1.54–2.50)**	2.10 (1.64–2.68)**
	2009	5.09 (4.15–6.25)**	2.96 (2.30–3.80)**	3.05 (2.37–3.92)**
	2010	5.11 (4.10–6.36)**	2.87 (2.18–3.76)**	2.89 (2.20–3.79)**
	2011	5.12 (4.19–6.26)**	2.99 (2.28–3.92)**	2.99 (2.28–3.93)**
	2012	6.56 (5.37–8.04)**	2.86 (2.09–3.90)**	2.86 (2.10–3.91)**
	2013	9.77 (7.92–12.03)**	3.86 (2.78–5.36)**	3.81 (2.74–5.29)**
	2014	9.57 (7.41–12.34)**	3.86 (2.67–5.59)**	3.79 (2.62–5.48)**
*Health Centre Characteristics*				
Location X duration of CQI participation				
Non-remote	Baseline		1.00 (reference)	1.00 (reference)
	1–2 cycles		1.77 (1.40–2.24)**	1.71 (1.35–2.17)**
	≥3 cycles		2.13 (1.49–3.03)**	2.02 (1.42–2.89)**
Remote	Baseline		1.00 (reference)	1.00 (reference)
	1–2 cycles		1.36 (0.90–2.05)	1.35 (0.90–2.04)
	≥3 cycles		1.35 (0.84–2.15)	1.35 (0.85–2.16)
Governance	Community-Controlled		1.00 (reference)	1.00 (reference)
	Government operated		0.73 (0.48–1.11)	0.74 (0.49–1.13)
Service population	≤500		1.00 (reference)	1.00 (reference)
	501–999		1.01 (0.67–1.52)	1.01 (0.67–1.51)
	≥1000		0.72 (0.49–1.05)	0.71 (0.49–1.04)
% Clients attending in past 6 months	<97 %		1.00 (reference)	1.00 (reference)
	≥97 %		1.39 (1.25–1.55)**	1.38 (1.23–1.54)**
*Client Characteristics*				
Sex	Male			1.00 (reference)
	Female			1.02 (0.95–1.10)
Age	≥15- < 25			1.00 (reference)
	≥25- < 40			1.09 (0.81–1.47)
	≥40- < 55			1.43(1.06–1.92)*
	≥55			1.52 (1.13–2.05)**
Indigenous Status	Non-Indigenous			1.00 (reference)
	Indigenous			1.27 (1.06–1.51)**
Comorbidity	None			1.00 (reference)
	One or more			1.31 (1.19–1.44)**
Complications	No complications			1.00 (reference)
	1–2 complications			1.16 (1.03–1.30)*
	>2 complications			0.90 (0.74–1.09)
Random effects (intercepts)				
	State (variance (SE))	0.80 (0.55)	0.39 (0.28)	0.39 (0.28)
	MOR_STATE_	2.34	1.82	1.82
* PCV (% explained variance)*		-	*51 %*	*51 %*
Health Centre (variance (SE))		0.90 (0.12)	0.79 (0.11)	0.78 (0.11)
	MOR_HC_	2.47	2.34	2.32
* PCV (% explained variance)*		-	*12 %*	*14 %*
State & Health Centre (variance)		1.70	1.19	1.17
	MOR_STATE-HC_	3.47	2.83	2.81
* PCV (% explained variance)*		-	*30 %*	*31 %*
	Patient (variance (SE))	0.11 (0.05)	0.14 (0.05)	0.07 (0.03)

MOR (Median odds ratio): odds of receiving ‘top quartile’ service delivery if patient was to change health centre or jurisdiction; PCV (Proportional change in variance): percent variation explained in odds for better health care delivery by introduction of health centre or patient level factors

*p < 0.05

**p = <0.01

**Table 7:** Adjusted multilevel logistic regression models of health centre and client level factors associated with top quartile delivery of guideline-scheduled specialist physical checks (*N* = 15,622 clients, 162 health centres)

		Model A empty	Model B adjusted	Model C adjusted
	Fixed effects	OR (95 % CI)	ORs (95 % CI)	ORs (95 % CI)
Audit Year	2005/2006	1.00 (reference)	1.00 (reference)	1.00 (reference)
	2007	0.88 (0.72–1.08)	0.74 (0.60–0.91)**	0.75 (0.60–0.93)**
	2008	0.95 (0.80–1.13)	0.78 (0.63–0.96)*	0.83 (0.67–1.03)
	2009	1.26 (1.04–1.51)*	1.02 (0.82–1.27)	1.05 (0.84–1.32)
	2010	1.06 (0.87–1.29)	0.86 (0.68–1.09)	0.86 (0.68–1.10)
	2011	1.02 (0.85–1.21)	0.87 (0.69–1.10)	0.86 (0.67–1.09)
	2012	1.29 (1.08–1.53)*	0.95 (0.73–1.24)	0.95 (0.72–1.25)
	2013	1.64 (1.36–1.97)**	1.16 (0.87–1.54)	1.09 (0.82–1.46)
	2014	1.65 (1.30–2.08)**	1.21 (0.87–1.68)	1.13 (0.81–1.57)
*Health Centre Characteristics*				
Location X duration of CQI participation				
Non-remote	Baseline		1.00 (reference)	1.00 (reference)
	1–2 cycles		1.59 (1.32–1.91)**	1.53 (1.26–1.84)**
	≥3 cycles		1.26 (0.93–1.71)	1.20 (0.88–1.65)
Remote	Baseline		1.00 (reference)	1.00 (reference)
	1–2 cycles		1.24 (0.91–1.68)	1.36 (0.99–1.85)
	≥3 cycles		1.55 (1.07–2.24)*	1.70 (1.16–2.47)**
Governance	Community-Controlled		1.00 (reference)	1.00 (reference)
	Government operated		0.89 (0.66–1.20)	0.93 (0.68–1.26)
Service population	≤500		1.00 (reference)	1.00 (reference)
	501–999		0.94 (0.70–1.26)	0.94 (0.70–1.27)
	≥1000		0.86 (0.65–1.12)	0.86 (0.65–1.14)
% Clients attending in past 6 months	<97 %		1.00 (reference)	1.00 (reference)
	≥97 %		1.47 (1.32–1.64)**	1.46 (1.31–1.62)**
*Client Characteristics*				
Sex	Male			1.00 (reference)
	Female			1.14 (1.06–1.23)**
Age	≥15- < 25			1.00 (reference)
	≥25- < 40			1.40 (1.07–1.83)*
	≥40- < 55			1.92 (1.48–2.51)**
	≥55			2.66 (2.04–3.47)**
Indigenous Status	Non-Indigenous			1.00 (reference)
	Indigenous			1.23 (1.05–1.45)*
Comorbidity	None			1.00 (reference)
	One or more			1.35 (1.24–1.47)**
Disease severity	No complications			1.00 (reference)
	1–2 complications			2.34 (2.06–2.67)**
	>2 complications			3.08 (2.45–3.88)**
Random effects (intercepts)				
	State (variance (SE))	0.36 (0.26)	0.18 (0.14)	0.18 (0.14)
	MOR_STATE_	1.77	1.49	1.49
* PCV (% explained variance)*		-	*49 %*	*51 %*
Health Centre (variance (SE))		0.49 (0.07)	0.38 (0.06)	0.40 (0.06)
	MOR_HC_	1.95	1.80	1.83
* PCV (% explained variance)*		-	*23 %*	*19 %*
State & Health Centre (variance)		0.85	0.56	0.57
	MOR_STATE-HC_	2.41	2.03	2.06
* PCV (% explained variance)*		-	*34 %*	*32 %*
	Patient (variance (SE))	1.21 (0.35)	1.00 (0.27)	0.26 (0.08)

MOR (Median odds ratio): odds of receiving ‘top quartile’ service delivery if patient was to change health centre or jurisdiction; PCV (Proportional change in variance): percent variation explained in odds for better health care delivery by introduction of health centre or patient level factors

*p < 0.05

**p = <0.01

**Table 8:** Adjusted multilevel logistic regression models of health centre and client level factors associated with top quartile delivery of guideline-scheduled counselling or education (for nutrition and physical activity) (*N* = 15,622 clients, 162 health centres

		Model A empty	Model B adjusted	Model C adjusted
	Fixed effects	OR (95 % CI)	ORs (95 % CI)	ORs (95 % CI)
Audit Year	2005/2006	1.00 (reference)	1.00 (reference)	1.00 (reference)
	2007	1.68 (1.38–2.04)**	1.27 (1.03–1.57)*	1.33 (1.08–1.64)**
	2008	1.69 (1.41–2.01)**	1.36 (1.11–1.68)**	1.46 (1.18–1.80)**
	2009	2.69 (2.24–3.23)**	2.20 (1.76–2.74)**	2.26 (1.81–2.82)**
	2010	2.69 (2.22–3.26)**	2.37 (1.82–3.00)**	2.40 (1.89–3.03)**
	2011	2.14 (1.80–2.54)**	2.06 (1.62–2.61)**	2.06 (1.62–2.61)**
	2012	2.18 (1.83–2.59)**	1.67 (1.27–2.18)**	1.64 (1.26–2.15)**
	2013	2.43 (2.86–4.12)**	2.58 (1.93–3.44)**	2.52 (1.89–3.36)**
	2014	2.48 (1.98–3.12)**	2.13 (1.54–2.96)**	2.10 (1.51–2.91)**
*Health Centre Characteristics*				
Location X duration of CQI participation				
Non-remote	Baseline		1.00 (reference)	1.00 (reference)
	1–2 cycles		2.09 (1.72–2.53)**	2.04 (1.68–2.47)**
	≥3 cycles		0.92 (0.68–1.25)	0.90 (0.66–1.22)
Remote	Baseline		1.00 (reference)	1.00 (reference)
	1–2 cycles		1.27 (0.84–1.91)	1.31 (0.91–1.87)
	≥3 cycles		2.41 (1.54–3.79)**	2.47 (1.64–3.74)**
Governance	Community-Controlled		1.00 (reference)	1.00 (reference)
	Government operated		0.85 (0.59–1.20)	0.87 (0.62–1.22)
Service population	≤500		1.00 (reference)	1.00 (reference)
	501–999		0.96 (0.66–1.41)	0.95 (0.65–1.39)
	≥1000		0.80 (0.56–1.14)	0.81 (0.57–1.14)
% Clients attending in past 6 months	<97 %		1.00 (reference)	1.00 (reference)
	≥97 %		1.30 (1.17–1.44)**	1.29 (1.16–1.44)**
*Client Characteristics*				
Sex	Male			1.00 (reference)
	Female			1.02 (0.95–1.09)
Age	≥15- < 25			1.00 (reference)
	≥25- < 40			1.29 (0.99–1.70)
	≥40- < 55			1.41 (1.08–1.85)*
	≥55			1.36 (1.04–1.78)*
Indigenous Status	Non-Indigenous			1.00 (reference)
	Indigenous			0.97 (0.83–1.14)
Comorbidity	None			1.00 (reference)
	One or more			1.38 (1.27–1.51)**
Complications	No complications			1.00 (reference)
	1–2 complications			1.17 (1.05–1.31)**
	>2 complications			1.03 (0.86–1.24)
Random effects (intercepts)				
	State (variance (SE))	0.12 (0.12)	0.002 (0.02)	0.0002 (0.005)
	MOR_STATE_	1.38	1.04	1.01
* PCV (% explained variance)*		-	*98 %*	*100 %*
Health Centre (variance (SE))		0.77 (0.11)	0.69 (0.10)	0.68 (0.09)
	MOR_HC_	2.31	2.21	2.20
* PCV (% explained variance)*		-	*10 %*	*12 %*
State & Health Centre (variance)		0.88	0.70	0.68
	MOR_STATE-HC_	2.45	2.22	2.20
* PCV (% explained variance)*		-	*21 %*	*23 %*
	Patient (variance (SE))	0.62 (0.12)	0.47 (0.12)	0.27 (0.08)

MOR (Median odds ratio): odds of receiving ‘top quartile’ service delivery if patient was to change health centre or jurisdiction; PCV (Proportional change in variance): percent variation explained in odds for better health care delivery by introduction of health centre or patient level factors

*p < 0.05

**p = <0.01

**Table 9:** Adjusted multilevel logistic regression models of health centre and client level factors associated with top quartile delivery of guideline-scheduled counselling or education (for smoking and alcohol risk reduction amongst high risk users) (*N* = 5496 clients, 162 health centres)

		Model A empty	Model B adjusted	Model C adjusted
	Fixed effects	OR (95 % CI)	ORs (95 % CI)	ORs (95 % CI)
Audit Year	2005/2006	1.00 (reference)	1.00 (reference)	1.00 (reference)
	2007	3.30 (2.23–4.88)**	2.67 (1.77–4.03)**	2.80 (1.85–4.22)**
	2008	2.32 (1.67–3.21)**	1.87 (1.31–2.67)**	1.96 (1.37–2.81)**
	2009	2.33 (1.71–3.18)**	1.90 (1.34–2.69)**	1.96 (1.38–2.78)**
	2010	2.21 (1.59–3.09)**	1.88 (1.30–2.74)**	1.90 (1.31–2.76)**
	2011	2.25 (1.66–3.05)**	1.99 (1.41–2.82)**	2.01 (1.42–2.85)**
	2012	2.25 (1.67–3.02)**	1.74 (1.19–2.54)**	1.75 (1.20–2.56)**
	2013	2.76 (2.03–3.76)**	2.06 (1.39–3.06)**	2.07 (1.40–3.07)**
	2014	2.41 (1.67–3.45)**	1.81 (1.15–2.86)*	1.82 (1.16–2.88)**
*Health Centre Characteristics*				
Location X duration of CQI participation				
Non-remote	Baseline		1.00 (reference)	1.00 (reference)
	1–2 cycles		1.83 (1.29–2.59)**	1.78 (1.26–2.53)**
	≥3 cycles		1.71 (1.01–2.89)*	1.64 (0.97–2.77)
Remote	Baseline		1.00 (reference)	1.00 (reference)
	1–2 cycles		0.94 (0.65–1.35)	0.93 (0.65–1.35)
	≥3 cycles		1.03 (0.61–1.75)	1.04 (0.62–1.76)
Governance	Community-Controlled		1.00 (reference)	1.00 (reference)
	Government operated		0.67 (0.50–0.91)*	0.69 (0.51–0.94)*
Service population	≤500		1.00 (reference)	1.00 (reference)
	501–999		0.92 (0.67–1.27)	0.92 (0.66–1.27)
	≥1000		0.99 (0.74–1.34)	1.00 (0.74–1.35)
% Clients attending in past 6 months	<97 %		1.00 (reference)	1.00 (reference)
	≥97 %		1.06 (0.90–1.26)	1.05 (0.88–1.24)
*Client Characteristics*				
Sex	Male			1.00 (reference)
	Female			1.00 (0.88–1.13)
Age	≥15- < 25			1.00 (reference)
	≥25- < 40			0.90 (0.57–1.42)
	≥40- < 55			0.97 (0.62–1.53)
	≥55			0.92 (0.58–1.45)
Indigenous Status	Non-Indigenous			1.00 (reference)
	Indigenous			1.07 (0.76–1.51)
Comorbidity	None			1.00 (reference)
	One or more			1.23 (1.06–1.43)**
Disease severity	No complications			1.00 (reference)
	1–2 complications			1.10 (0.90–1.35)
	>2 complications			1.17 (0.84–1.64)
Random effects (intercepts)				
	State (variance (SE))	0.01 (0.02)	0.00007 (0.001)	0.00008 (0.001)
	MOR_STATE_	1.09	1.01	1.01
* PCV (% explained variance)*		-	*99 %*	*99 %*
Health Centre (variance (SE))		0.44 (0.08)	0.35 (0.07)	0.35 (0.07)
	MOR_HC_	1.88	1.76	1.75
* PCV (% explained variance)*		-	*20 %*	*21 %*
State & Health Centre (variance (SE))		0.44	0.35	0.35
	MOR_STATE-HC_	1.89	1.76	1.75
* PCV (% explained variance)*		-	*20 %*	*22 %*
	Patient (variance (SE))	1.11 (0.16)	1.13 (0.27)	0.97 (0.35)

MOR (Median odds ratio): odds of receiving ‘top quartile’ service delivery if patient was to change health centre or jurisdiction; PCV (Proportional change in variance): percent variation explained in odds for better health care delivery by introduction of health centre or patient level factors

*p < 0.05

**p = <0.01

Health centre factors significantly associated with higher delivery were broadly similar across the modes of care. The unadjusted analyses showed that health centres with longer duration of participation in CQI had better odds of being within the top quartile for all modes of care. There was an interaction between health centre duration of participation in CQI and location (remote/non-remotely located). Inclusion of the interaction terms in the adjusted models showed that for laboratory tests, longer duration of participation in CQI was associated with higher delivery for remote health centres, but not for non-remote centres (Table 5). For the other modes of care, effects of CQI were evident for both remote and non-remote centres, but stronger effects were evident for remote centres across most of the modes (Tables 6, 7 and 9). Delivery of generalist physical checks was the only mode of care that showed consistent improvement with longer duration of CQI beyond 3 cycles of participation, for remote and non-remote health centres. Health centres with a higher proportion of patients that attended within the 6 months prior to audit had better odds of being within the top quartile for all modes of care except for delivery of counselling/education for high risk substance use.

The addition of patient level factors in Model C had modest effects, accounting for less than 1–2 % of the variation in service delivery across health centres and jurisdictions. Patient age, level of co-morbidity and number of disease complications were significantly associated with improved delivery across most modes of care (Tables 5, 6, 7, 8 and 9). Aboriginal and Torres Strait Islander identified patients were more likely to receive generalist physical checks than non-Aboriginal patients (Table 6), but this characteristic was not associated with increased odds of being within the top quartile of delivery for any other mode of care.

## Discussion

Our study found wide variation between health centres in delivery of different modes of care and different patterns of improvement over time. Very few previous studies have been able to examine service delivery over several years of participation in a CQI program as we have done in this study. In our study, health centres with longer participation in CQI had increased odds of being within the top quartile of delivery for all modes of care, and we echo the point made in our previous paper [1] that sustained commitment to CQI is required to realise and demonstrate improvement. Different trajectories of improvement during CQI participation for the different modes of care suggest areas where higher level system development may be required to achieve large scale change.

In the present study, the effect of audit year on service delivery was particularly marked for generalist physical checks relative to other modes of care. The inclusion of health centre level factors, including duration of participation in CQI moderated the effect of audit year, but associations between audit year and service delivery remained significant for this mode of care. We note the existence during this period of a range of system-wide initiatives to increase the uptake of Medicare reimbursed adult health assessments for Aboriginal and Torres Strait Islander adults (first introduced in 2004), including simplification of claiming processes for these checks, and additional workforce and other incentives that promoted their uptake [10, 11] The strong effect of audit year for delivery of generalist physical checks, relative to the other measures, and the higher delivery evident for Aboriginal and Torres Strait Islander clients, suggests that workforce and other initiatives in the broader system that promoted uptake of adult health assessments for Aboriginal and Torres Strait Islander adults may have contributed to the strong increase in delivery of generalist physical checks evident amongst the patients with Type 2 diabetes in our study.

Our findings in relation to the continued evidence-practice gaps evident for delivery of specialist-delivered checks in this large dataset raises questions about the extent to which recent initiatives to improve Aboriginal and Torres Strait Islander peoples’ access to specialists through PHC are integrated into PHC delivery and record- keeping systems and reaching those who need them. Although health centres with longer duration of CQI had higher odds of being in the top quartile of delivery for specialist-delivered checks, this is relative to a general low overall delivery in this area. To achieve improvements in delivery, health centres need access to specialists for their patients when and where required—this needs evidence-informed action at higher levels of the system, and a learning orientation to policy development and implementation. At the minimum, solutions to improve delivery of specialist checks in the Aboriginal and Torres Strait Islander PHC context are likely to include a greater focus on improved integration of visiting services into PHC delivery systems—this may for example, include placing greater emphasis on active training of PHC staff by visiting Podiatrists, so that the PHC team can routinely check feet and identify problems, ensuring greater co-ordination and integration of the multiple eye health initiatives underway in Aboriginal and Torres Strait Islander communities, and ensuring that tele-health services, if provided, are appropriately documented in health centre records.

The effect of audit year on delivery of laboratory tests was inconsistent for different audit years relative to baseline, and our study which was not designed to test for time effects, does not allow definitive explanation. In the unadjusted analysis, increased odds of higher service delivery relative to baseline were evident for audit years from 2010 onwards. During the period of the study, there was increasing emphasis on providing point-of-care pathology testing as a way to overcome the challenges of providing pathology services to remote communities—with at least one new wide-scale point-of-care initiative implemented in Aboriginal and Torres Strait Islander medical services during the latter years of our study [12]. The finding that delivery of laboratory tests improved with participation in CQI in remote but not in non-remote health centres may reflect relatively greater use of multiple service providers in non-remote areas, and/or a relatively greater emphasis on using point-of-care pathology testing in remote areas.

The finding that our models were only able to explain a relatively small proportion of the variation between health centres in delivery of counselling and education for nutrition and physical activity and for high risk substance use (21 and 20 % respectively) may indicate a greater influence of factors not accounted for in our study in relation to these modes of care. For example, in relation to these more ‘non- technical’ modes of care, factors such as composition of PHC teams, allocation of work, and different policies and practices in relation to documentation of these care processes may be relatively more important than for some of the more technical or medical aspects of care where a larger proportion of variation was explained by the models. One of the few previous multi-level studies examining variation in delivery of interventions for lifestyle risk factors in PHC [13] explained over 80 % of variance between providers, noting that the team in which the providers worked and beliefs about perceived effectiveness and accessibility of support services, were most important in determining delivery of counselling/education type interventions in relation to lifestyle risk factors. Our study did not measure factors such as team composition and accessibility of support services. Previous research conducted in a relatively well-resourced remote health centre in the Northern Territory estimated that funding levels were inadequate for delivery of the full scope of services scheduled in guidelines for people with type 2 diabetes and chronic kidney disease in this community [14]—in a context of under-funding for implementing all best practice guidelines for chronic illness care, it may be that health centres prioritise which types of care processes they offer to clients with chronic disease.

Particular strengths of our study were the measurement of a broad range of service delivery indicators based on best practice clinical guidelines over a number of years of CQI implementation, the ability to correct for a range of confounding factors, and the ability to consider the effects of audit year alongside, and independent of the effects of CQI participation (owing to differing time periods of enrolment in the study). There were also a number of limitations. Health centres were not randomly selected and their participation in the project was on a voluntary basis, which limits generalizability of the findings. In particular, there were fewer health centres included in 2014 compared to other years. Our data on delivery of care processes were obtained from clinical records, which may underestimate service delivery due to under-documentation—and we recognise that the extent of documentation may differ systematically by mode of care. However, given that delivering services to patients with chronic illness is a periodic and on-going process involving multiple service providers, accurate and clear documentation of services delivered is a critical aspect of quality.

## Conclusions

The findings of this study, showing different levels of delivery and patterns of improvement for different modes of care over time, suggest that achieving large scale improvement in all areas of recommended care is likely to require commitment to identifying evidence-practice gaps in different areas of care across different localities and using this evidence to inform action at various levels of the health system.

Our findings of an association between CQI participation and higher delivery for all modes of care suggests that CQI at health centre level has the potential to improve care delivery across the scope of services—but acting on its own, without higher level support, the effects achieved may remain modest and, for some modes of care, may not be sustained. Our findings are a reminder that actions at different levels of the system are likely to have an important influence on the nature and scale of improvements that are achieved.

Analysis of delivery of services based on how care is delivered shows promise for identifying areas of systems requiring further development and for encouraging a focus on finding solutions to problems of care quality at different levels of the system. We recommend further exploration of the utility of different forms of analysis of aggregate CQI data for large scale improvement. Such analysis may also contribute to understanding system response to policy and funding levers. We caution policy makers and service managers against looking to unadjusted trends in service delivery as a simple barometer of success of CQI, noting the range of contextual factors that can influence service delivery over time—the primary use of aggregate CQI data should be to guide improvement.

## Abbreviations

CI: Confidence Interval
CQI: Continuous Quality Improvement
PHC: Primary health care
PCV: Proportional change in variance
MOR: Median odds ratio
